# The impact of tacrolimus variability on first year renal function in kidney transplant recipients: A retrospective cohort study

**DOI:** 10.1097/MD.0000000000050881

**Published:** 2026-10-02

**Authors:** Ferah Taran, Ercan Türkmen

**Affiliations:** a Department of Nephrology, Şirnak State Hospital, Şirnak, Turkey; b Department of Nephrology, Faculty of Medicine, Ondokuz Mayis University, Samsun, Turkey.

**Keywords:** eGFR, immunosuppression, kidney transplantation, tacrolimus variability, therapeutic drug monitoring

## Abstract

Tacrolimus is a key immunosuppressant with a narrow therapeutic range in kidney transplantation. Early fluctuations in trough concentrations may influence graft stability, yet the clinical significance of early intrapatient variability remains uncertain. This study aimed to evaluate whether tacrolimus variability during the first posttransplant month is associated with renal function at 12 months. This single-center retrospective cohort study included adult kidney transplant recipients receiving tacrolimus-based immunosuppression between January 2017 and December 2021. Tacrolimus variability was quantified as the absolute difference (Δ, ng/mL) between maximum and minimum trough concentrations during the first 30 days, classified as high (Δ > 8 ng/mL) or low (Δ ≤ 8 ng/mL). Renal function was assessed using eGFR at 1, 3, 6, and 12 months. Statistical analyses applied appropriate parametric and nonparametric tests with a significance level of *P* < .05. High variability was associated with more frequent subtherapeutic and supratherapeutic trough concentrations and transient reductions in early graft performance. However, 12-month eGFR was not significantly different between variability groups. In multivariable models, recipient age, donor age, and biopsy-proven graft dysfunction were consistently associated with lower eGFR across follow-up. Male donor sex showed a positive association with eGFR only at 6 months, whereas early tacrolimus variability was not independently associated with eGFR at any time point.

## 1. Introduction

Tacrolimus is the primary immunosuppressant used in kidney transplantation, with a narrow therapeutic range and known high inter- and intrapatient pharmacokinetic variability.^[1,2]^ As a consequence of this variability, recipients may experience excessive or subtherapeutic drug exposure; as a result, adequate immunosuppression may not be achieved, or toxicity may develop.^[3]^ Some clinical observational studies have demonstrated a relationship between high intrapatient variability (IPV) and deterioration in kidney function, including reduced estimated glomerular filtration rate (eGFR).^[4-8]^

The variability in the evaluation steps for IPV calculation – including measurement duration, statistical variables used, and threshold value determination criteria – varies across studies, which hinders the establishment of a unified interpretation across the literature. While coefficient of variation (CV) or standard deviation have traditionally been used,^[9-11]^ recent studies have defined new metrics such as the “tacrolimus variability score.”^[1,9,12-15]^ The measurement period also varies: Kim et al used the CV of tacrolimus levels over 6 to 12 months as IPV,^[2]^ while Park et al evaluated using time-weighted CV over the first 2 years.^[16]^ Additionally, some studies performed immunological risk subgroup analyses (e.g., PRA positivity), while others did not.^[2,16-20]^ No unified conclusions have been drawn regarding the clinical significance of IPV.^[14]^ For example, while statistically significant differences have been reported between Tac-IPV values calculated in the first year posttransplantation and renal function markers, improved eGFR has been observed in patients with low variability (LV) in the long term.^[6,21,22]^ The lack of subgroup analyses and uncertainty regarding the statistical and clinical significance of effect sizes also perpetuate ongoing debates in the field.^[6,23]^ Additionally, factors such as the frequency of blood sampling, dose adjustments, or drug interactions may also influence IPV calculations.^[24]^

To examine variability in drug levels more systematically, different definitions of Tac-IPV were compared. The associations between off-target drug concentrations and alternative IPV definitions were examined within this scope. Specifically, using 2 separate IPV metrics, the relationships between 12-month eGFR values and deviations from target drug levels were analyzed. The design is retrospective and observational in nature; analyses were based on retrospective data from a defined cohort of renal transplant recipients. This approach aims to support decision-making processes by identifying which IPV definition may offer greater utility in guiding patient monitoring and immunosuppressive strategy.

## 2. Methods and materials

### 2.1. Study design and ethical approval

This single-center retrospective cohort study included adult kidney transplant recipients who underwent transplantation and follow-up at Ondokuz Mayis University Hospital between January 2017 and December 2021. Clinical and laboratory data were extracted from the institutional electronic medical record system of Ondokuz Mayis University Hospital, where the transplant follow-up records were generated and stored. Access to and use of the dataset were authorized by Ondokuz Mayis University Hospital as the data-holding institution through institutional permission. All records were de-identified before transfer and analysis, and only anonymized study IDs were used. The Health Sciences University Gazi Yasargil Training and Research Hospital Ethics Committee reviewed and approved the investigator-led retrospective analysis protocol for the secondary use of anonymized clinical records (Approval No: 458, dated April 25,2025). The study was conducted in accordance with the Declaration of Helsinki.

### 2.2. Participant selection

The study population consisted of adult renal transplant recipients managed with tacrolimus-based immunosuppressive therapy during the first 12 months posttransplant (n = [167] patients). Eligible cases were retrospectively identified via the institutional digital health records system (Nucleus). Inclusion criteria required patients to be ≥18 years of age, to have completed at least 12 months of follow-up, to have ≥3 tacrolimus trough level measurements within the first 30 days, and to have received a consistent tacrolimus formulation throughout the follow-up. Exclusion criteria included combined organ transplantation, death within the first year, incomplete or missing medical records, use of an alternative calcineurin inhibitor (e.g., cyclosporine), and documented medication nonadherence (e.g., off-prescription use or irregular follow-up). These criteria were designed to ensure a homogeneous cohort suitable for evaluating pharmacokinetic variability and its clinical correlates.

As this was a retrospective cohort study, all eligible kidney transplant recipients who met the inclusion criteria during the study period were included. Therefore, no a priori sample-size calculation was performed. The full-cohort approach maximized statistical power and minimized selection bias.

A participant-flow diagram summarizing screening, exclusions, and the final analytic cohort is presented in Figure 1.

**Figure 1.**
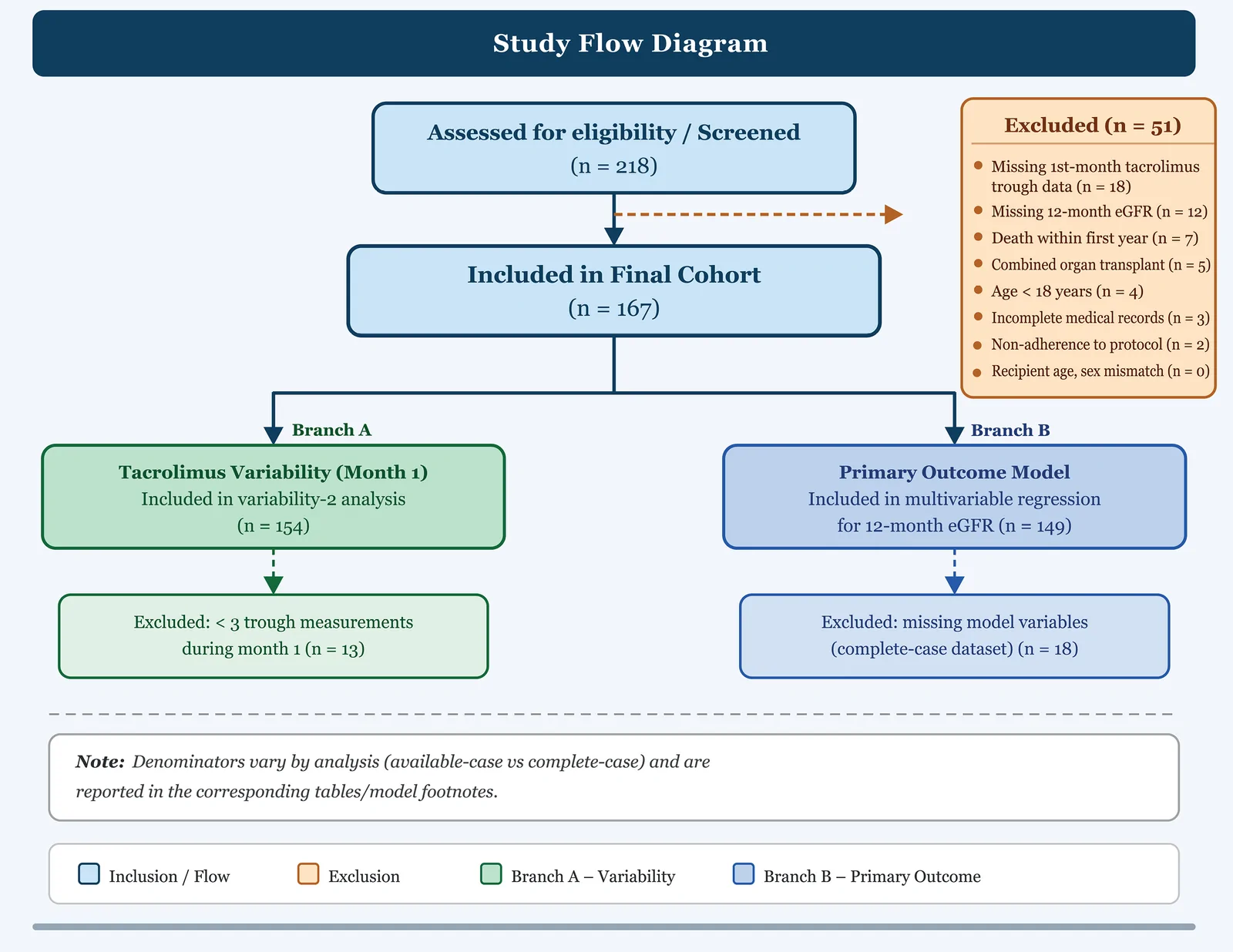
Participant-flow diagram summarizing screening, exclusions, and the final analytic cohort. eGFR = estimated glomerular filtration rate.

### 2.3. Definitions and variables

Tacrolimus variability during the first posttransplant month was assessed using 1 primary quantitative definition and 1 descriptive exposure pattern. First, a descriptive classification based on trough concentration patterns was recorded to characterize exposure trends: subtherapeutic (<6 ng/mL), target (6–10 ng/mL), and supratherapeutic (>10 ng/mL). For exploratory analyses, subtherapeutic and supratherapeutic profiles were combined as “off-target,” while target and supratherapeutic profiles were combined as “target-high.” Second and primary, tacrolimus variability was quantified as the absolute difference (Δ, ng/mL) between the maximum and minimum trough levels obtained during the first 30 days. High variability was defined as Δ > 8 ng/mL and LV as Δ ≤ 8 ng/mL. Off-target concentrations were defined as <6 ng/mL or >10 ng/mL. Renal function was evaluated using the CKD-EPI–based eGFR.

### 2.4. Outcome measures

The primary outcome of the study was renal function at 12 months posttransplantation, assessed using eGFR calculated with the CKD-EPI equation. Secondary outcomes included renal function at 1, 3, and 6 months; serum creatinine at corresponding time points; and the number of off-target tacrolimus trough levels (<6 ng/mL or >10 ng/mL) during the first month. All outcomes were evaluated within predefined follow-up time windows.

### 2.5. Data collection

All relevant data were extracted from electronic medical records and independently verified by 2 researchers. Collected variables included demographic characteristics, clinical indicators (donor type, rejection history, delayed graft function [DGF], comorbidities), and laboratory measurements (tacrolimus concentrations, serum creatinine, and eGFR). Tacrolimus monitoring data were processed using a standardized cleaning procedure. A trough level was defined as a sample drawn within 60 minutes before the scheduled morning dose, and when repeated measurements occurred on the same day, only the value closest to the trough time was retained. Random (non-trough) samples and import-related duplicate entries were removed to ensure that only valid through concentrations were included in variability analyses. Renal function indicators were collected at predefined time points (1, 3, 6, and 12 months), and all cleaned data were used consistently across descriptive, comparative, and multivariable analyses.

### 2.6. Statistical analysis

Continuous variables were assessed for normality using Shapiro–Wilk tests and visual methods (histograms and *Q–Q* plots). For group comparisons, parametric tests were used when assumptions were met; otherwise, nonparametric alternatives were applied. Categorical variables were compared using χ^2^ tests or Fisher’s exact tests where appropriate. For multivariable analyses, predictors were selected based on clinical relevance and prior literature, with additional inclusion of variables showing a univariate association with *P* < .20. Linear regression assumptions were evaluated through residual diagnostics, including assessments of homoscedasticity, normality, multicollinearity (variance inflation factors), and influential observations (Cook’s distance). Due to non-normal residual distributions, robust regression was applied for the 3- and 6-month eGFR models. Nonlinearity between continuous predictors and eGFR was examined using LOWESS smoothing; no substantial deviation from linearity was observed. Missing data were evaluated at both the variable and model levels. Although missingness was low for individual variables, complete-case restriction in multivariable analyses resulted in lower analytic denominators than the full eligible cohort. Therefore, multivariable models were fitted using complete cases, and the analytic sample size for each model was reported in the corresponding table note. Sensitivity analyses using alternative tacrolimus variability thresholds (Δ ≥ 6 ng/mL and Δ ≥ 10 ng/mL) produced results consistent with the primary models. Because analyses were exploratory and effect sizes were emphasized, no formal correction for multiple comparisons was applied. Statistical significance was defined as *P* < .05 (2-sided).

## 3. Results

A total of 167 kidney transplant recipients met the eligibility criteria and were included in the study. A participant-flow diagram summarizing patient screening, exclusions, and the final analytic sample is provided in Figure 1.

Table 1 summarizes baseline characteristics reported as n (%), including demographic features, clinical indicators, transplant-related variables, and immunosuppressive profiles. Most recipients were male (58.1%) and received kidneys from deceased donors (59.9%). Biopsy-proven graft dysfunction occurred in 44.9% of recipients, and biopsy-proven acute rejection was observed in 19.2% of the overall cohort. For tacrolimus variability-2, high IPV was defined as an absolute maximum–minimum trough difference during month 1 (Δ) > 8 ng/mL and was present in 83.8% of recipients.

**Table 1:** Frequency distributions of baseline demographic, clinical, and transplant-related categorical variables in renal transplant recipients.

Variable	n (%)
Recipient sex	
Female	70 (41.9)
Male	97 (58.1)
Tacrolimus variability-1	
Normal–high	48 (28.7)
Low–high	119 (71.3)
Tacrolimus variability-2*	
LV (Δ ≤ 8 ng/mL)	27 (16.2)
HV (Δ > 8 ng/mL)	140 (83.8)
Delayed graft function	
No	141 (84.4)
Yes	26 (15.6)
Biopsy-proven graft dysfunction	
No	92 (55.1)
Yes	75 (44.9)
Biopsy diagnosis	
Minimal change	20 (31.3)
CNI toxicity	11 (17.2)
Acute rejection	30 (46.9)
Other†	3 (4.7)
Acute rejection (biopsy proven)	
No	135 (80.8)
Yes	32 (19.2)
Rejection phenotype	
ABMR	5 (15.6)
TCMR	14 (43.8)
Borderline	7 (21.9)
Mixed	6 (18.8)
Graft loss (return to dialysis)	9 (5.4)
Death	6 (3.6)
Primary kidney disease	
Diabetes mellitus	14 (8.4)
Hypertension	44 (26.3)
Glomerulonephritis	23 (13.8)
Other	40 (24.0)
Unknown	46 (27.5)
Comorbidities	
Diabetes present	16 (9.6)
Hypertension present	98 (58.7)
Obesity present	15 (9.0)
Recipient serology	
Anti-HCV positivity	3 (1.8)
CMV IgG positivity	158 (94.6)
EBV IgG positivity	155 (93.4)
Transplant number	9 (5.4) second or third graft
HLA mismatches	median 5 (range 2–6)
Desensitisation	9 (5.4)
Donor type	67 (40.1) living
Donor sex	88 (52.7) male
Tacrolimus formulation at baseline	129 (77.2) extended-release

GN = glomerulonephritis, HV = high variability, LV = low variability.

* Δ indicates the absolute difference between the maximum and minimum tacrolimus trough concentrations during month 1 (ng/mL). High variability was defined as Δ > 8 ng/mL.

† Acute interstitial nephritis = 1, GN recurrence = 2.

Table 2 summarizes continuous clinical and pharmacological variables, reported as mean ± standard deviation and median (minimum–maximum). Parameters include recipient and donor age, dialysis duration, tacrolimus exposure metrics, and renal function indicators (eGFR [mL/min/1.73 m^2^] and serum creatinine [mg/dL]) at the 1st, 3rd, 6th, and 12th months posttransplantation. The median recipient age was 44.1 years, and the median pretransplant dialysis duration was 48 months. Early posttransplant kidney function demonstrated satisfactory graft performance, with a median eGFR of 77.4 mL/min/1.73 m^2^ at the 12th month. Tacrolimus exposure during the first month was highly variable, with a mean absolute fluctuation (maximum–minimum difference) of 13.53 ng/mL, and patients underwent a median of 10 tacrolimus trough level measurements during this period, reflecting intensive early therapeutic monitoring. As illustrated in Figures 2 and 3, variability groups exhibited comparable trends in eGFR across all time points.

**Table 2:** Descriptive statistics for continuous clinical and pharmacological parameters in renal transplant recipients.

Variable	Mean ± SD	Median (range)
Recipient age (yr)	43.71 ± 13.32	44.1 (17.9–92.5)
First-month tacrolimus samples (n)	10.64 ± 7.93	10 (3–18)
Samples < 6 ng/mL (n)	1.83 ± 1.60	2 (0–7)
Samples > 10 ng/mL (n)	4.35 ± 2.26	4 (0–10)
Samples in target range (n)	3.87 ± 2.01	4 (0–10)
Tacrolimus max–min absolute difference (ng/mL)	13.53 ± 8.59	11 (1–66)
Dialysis duration (mo)	67.10 ± 70.56	48 (0–384)
Donor age (yr)	42.41 ± 13.94	42 (12–68)
eGFR 1st month (mL/min/1.73 m^2^)	70.56 ± 30.69	68.4 (4.2–151.7)
eGFR 3rd month (mL/min/1.73 m^2^)	72.15 ± 29.02	69.5 (11.2–152.2)
eGFR 6th month (mL/min/1.73 m^2^)	78.27 ± 51.43	74.2 (5.3–152.2)
eGFR 12th month (mL/min/1.73 m^2^)	76.03 ± 27.97	77.4 (3.6–151.4)
Serum creatinine 1st month (mg/dL)	1.47 ± 1.18	1.20 (0.45–11.84)
Serum creatinine 3rd month (mg/dL)	1.36 ± 0.77	1.17 (0.46–5.70)
Serum creatinine 6th month (mg/dL)	1.35 ± 1.00	1.10 (0.43–9.84)
Serum creatinine 12th month (mg/dL)	1.38 ± 1.30	1.12 (0.47–11.75)

eGFR = estimated glomerular filtration rate, mo = month, SD = standard deviation.

**Figure 2.**
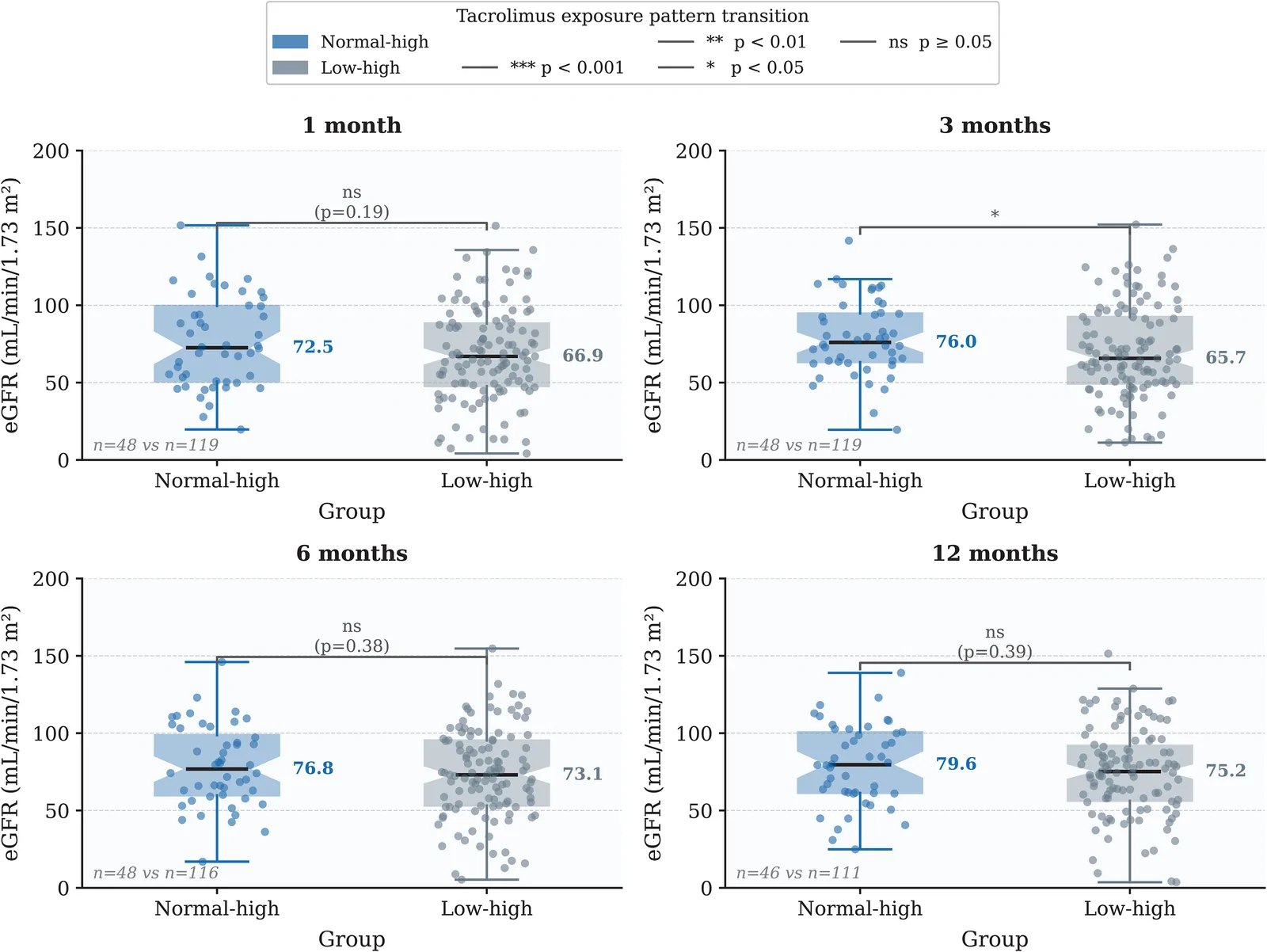
Distribution of eGFR values at 1, 3, 6, and 12 months according to tacrolimus exposure pattern transitions (normal–high vs low–high). eGFR = estimated glomerular filtration rate.

**Figure 3.**
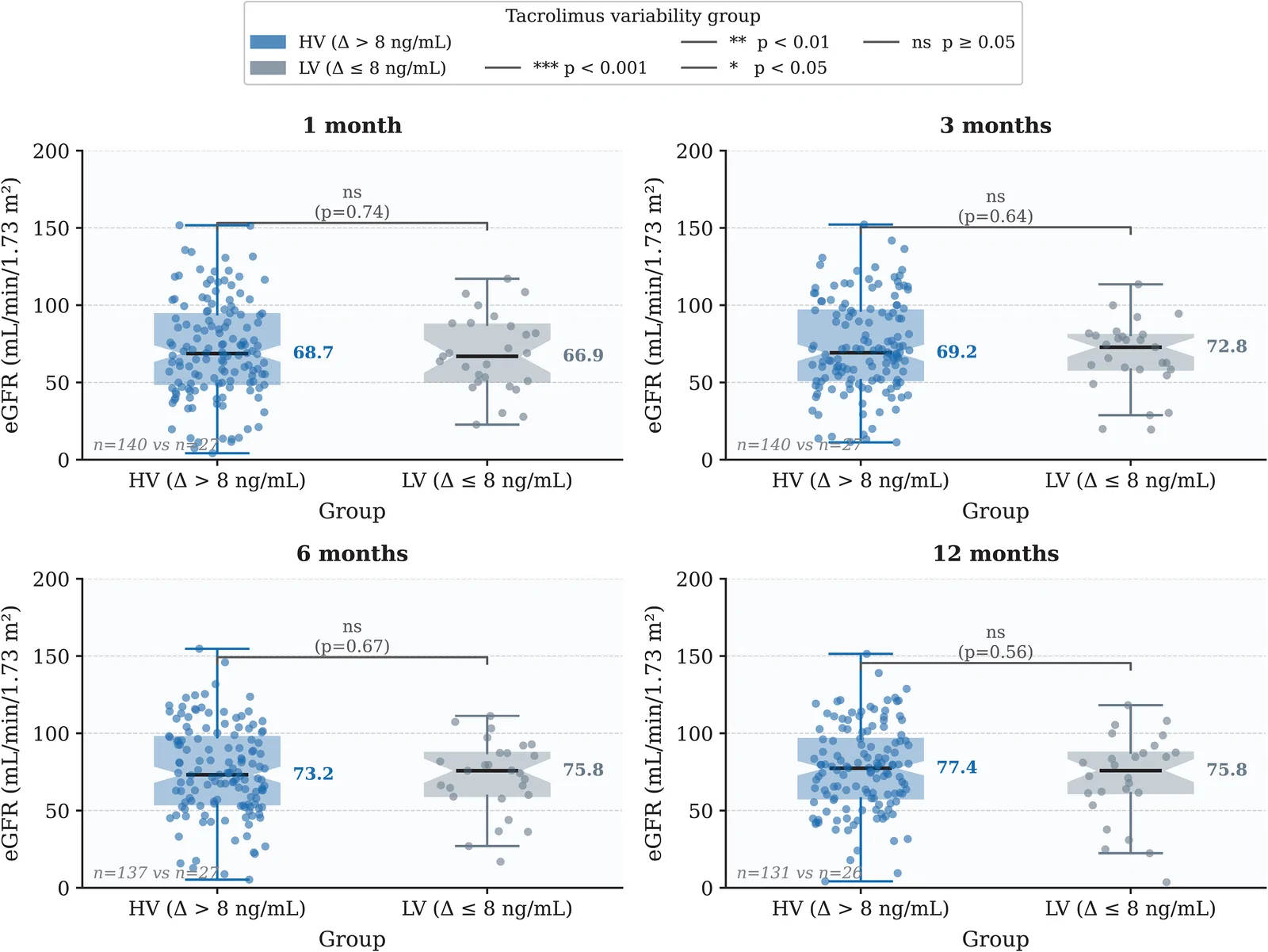
Distribution of eGFR values at 1, 3, 6, and 12 months in high- versus low-variability tacrolimus groups (Δ > 8 ng/mL vs Δ ≤ 8 ng/mL). eGFR = estimated glomerular filtration rate.

Table 3 displays selected quantitative variables compared between tacrolimus variability groups defined by 2 complementary approaches: a descriptive exposure pattern (variability 1) and the primary variability metric based on the absolute maximum–minimum trough difference (variability 2). Only variables with an effect size of at least Cohen’s *d* ≥ 0.20 are shown to ensure clinical relevance. Values are presented as mean ± standard deviation with medians in brackets. Group differences were tested using either independent-samples *t*-tests or Mann–Whitney *U* tests.

**Table 3:** Combined comparison of key quantitative variables by tacrolimus variability.

Variable	Variability 1 normal–high (n = 48) mean ± SD [Median]	Variability 1 low–high (n = 119) mean ± SD [Median]	*d*	*P*	Variability 2 low variability (Δ ≤ 8 ng/mL; n = 27) mean ± SD [Median]	Variability 2 high variability (Δ > 8 ng/mL; n = 140) mean ± SD [Median]	*d*	*P*
Renal function								
eGFR 1 mo (mL/min/1.73 m^2^)	76.2 ± 29.7 [72.6]	68.3 ± 30.9 [66.9]	0.26	.132*	—	—	—	—
eGFR 3 mo (mL/min/1.73 m^2^)	78.5 ± 24.4 [76.0]	69.6 ± 30.4 [65.7]	0.34	.031†	—	—	—	—
eGFR 12 mo (mL/min/1.73 m^2^)	—	—	—	—	70.9 ± 28.6 [75.9]	77.1 ± 27.9 [77.4]	–0.22	.307*
Demographics								
Recipient age (yr)	49.6 ± 13.9 [52.6]	49.5 ± 13.3 [49.1]	0.27	.087†	—	—	—	—
Donor age (yr)	39.5 ± 14.4 [39.5]	43.6 ± 13.7 [44.0]	–0.29	.089*	—	—	—	—
Tacrolimus exposure								
Samples < 6 ng/mL (count)	0.35 ± 1.21 [0]	2.42 ± 1.33 [2]	1.82	<.001†	1.22 ± 1.74 [0]	1.94 ± 1.55 [2]	0.40	.009†
Samples > 10 ng/mL (count)	4.79 ± 2.79 [5]	4.17 ± 1.99 [4]	0.22	.151†	2.81 ± 2.39 [2]	4.64 ± 2.12 [5]	0.61	<.001†
In-target samples (count)	—	—	—	—	4.70 ± 2.27 [5]	3.71 ± 1.93 [4]	0.32	.039†

Values are means ± standard deviations with medians in square brackets. “—” indicates that the variable did not meet the effect-size threshold for that variability definition. Only variables with Cohen’s *d* ≥ 0.20 are shown. Positive *d* indicates higher mean in the second group (low–high for variability 1; high variability for variability 2).

eGFR = estimated glomerular filtration rate, HV = high variability (Δ > 8 ng/mL), LV = low variability (Δ ≤ 8 ng/mL), SD = standard deviation.

* Independent-samples *t*-test.

† Mann–Whitney *U* test.

Across comparisons, higher tacrolimus variability was significantly associated with increased numbers of subtherapeutic (<6 ng/mL) and supratherapeutic (>10 ng/mL) trough levels under both definitions, with large effect sizes (*P* < .001; *d* up to 1.82). In Variability 2, patients classified as high variability (Δ > 8 ng/mL) also had significantly fewer in-target trough levels (*P* = .039; *d* = 0.32). In Variability 1, eGFR at 3 months showed a significant difference favoring the lower-variability exposure pattern (*P* = .031; *d* = 0.34). Other renal function parameters (eGFR at 1, 6, and 12 months), age, dialysis duration, and donor age did not differ significantly between groups (*P* > .05), despite small to moderate effect sizes.

Table 4 presents a combined cross-tabulation of categorical clinical variables associated with tacrolimus variability using the same 2 definitions. To focus on clinically relevant trends, variables demonstrating a statistical trend (*P* < .10) in at least 1 definition were included. Donor type was the only variable reaching significance under variability 2 (*P* = .022), with living donors being more common in the high-variability group (44.3%) compared with the low-variability group (18.5%). DGF approached significance in the variability 1 model (*P* = .061), where a higher frequency was observed among patients classified as low–high exposure pattern. Similarly, acute rejection demonstrated a borderline association under variability 2 (*P* = .099).

**Table 4:** Combined cross-tabulation of key categorical variables by tacrolimus variability.

Variable	Category	Variability 1 normal–high (n = 48)	Variability 1 low–high (n = 119)	*P*	Variability 2 low variability (Δ ≤ 8 ng/mL; n = 27)	Variability 2 high variability (Δ > 8 ng/mL; n = 140)	*P*
Delayed graft function	No	45 (93.8%)	96 (80.7%)	.061	24 (88.9%)	117 (83.6%)	.772
	Yes	3 (6.3%)	23 (19.3%)		3 (11.1%)	23 (16.4%)	
Acute-rejection phenotype	ABMR	1 (2.1%)	4 (3.4%)	.715	1 (3.7%)	4 (2.9%)	.099
	TCMR	3 (6.3%)	11 (9.2%)		0 (0.0%)	14 (10.0%)	
	Borderline	3 (6.3%)	4 (3.4%)		2 (7.4%)	5 (3.6%)	
	Mixed	1 (2.1%)	5 (4.2%)		0 (0.0%)	6 (4.3%)	
Donor type	Cadaveric	28 (58.3%)	72 (60.5%)	.933	22 (81.5%)	78 (55.7%)	.022
	Living	20 (41.7%)	47 (39.5%)		5 (18.5%)	62 (44.3%)	

Only variables with *P* < .10 in either definition are displayed. Yates-corrected χ^2^ tests were used unless otherwise specified; categories with small cell counts were analyzed using Fisher’s exact test. Multilevel rejection-phenotype categories were evaluated using Freeman–Halton exact procedures.

ABMR = antibody-mediated rejection, HV = high variability (Δ > 8 ng/mL), LV = low variability (Δ ≤ 8 ng/mL), TCMR = T-cell-mediated rejection.

Table 5 presents the results of multivariable regression analyses assessing the associations of clinical and pharmacological variables with eGFR at 1, 3, 6, and 12 months posttransplantation. The table displays unstandardized β coefficients for each independent variable. Regression models for 1- and 12-month eGFR were conducted using standard multiple linear regression, whereas robust regression was applied for the 3- and 6-month eGFR models because of non-normal model residuals. Recipient age, donor age, and biopsy-proven graft dysfunction showed consistent negative associations with eGFR across all follow-up time points. Male donor sex showed a positive association with eGFR at 6 months only. Living donor status and extended-release tacrolimus formulation were associated with eGFR only in the 1-month model and did not show consistent associations thereafter. Dialysis duration, recipient sex, DGF, acute rejection, and tacrolimus exposure variability were not significantly associated with eGFR in any model. Importantly, tacrolimus exposure variability, defined as an absolute maximum–minimum trough difference >8 ng/mL during the first month, did not show a statistically significant association with eGFR at any follow-up time point.

**Table 5:** Summary of multivariate regression analyses for predictors of estimated glomerular filtration rate (eGFR) at 1, 3, 6, and 12 months posttransplantation.

Predictor	1 Mo β	3 Mo β	6 Mo β	12 Mo β
Recipient age (yr)	−0.661***	−0.583***	−0.635***	−0.511**
Donor age (yr)	−0.417**	−0.546***	−0.694***	−0.684***
Dialysis duration (mo)	0.052 ns	−0.021 ns	0.035 ns	0.041 ns
Male recipient	−2.769 ns	−5.267 ns	0.078 ns	−0.330 ns
Delayed graft function	−6.527 ns	−9.115 ns	−3.853 ns	−3.013 ns
Biopsy-proven graft dysfunction	−18.921***	−24.245***	−22.902***	−21.271***
Acute rejection	−0.575 ns	−2.402 ns	−2.530 ns	−0.028 ns
Living donor (vs cadaveric)	11.922*	0.452 ns	1.567 ns	6.061 ns
Male donor	2.582 ns	4.216 ns	7.765*	6.657 ns
Extended-release tacrolimus (vs IR)	−13.117*	−3.813 ns	−4.916 ns	−3.072 ns
High variability (Δ > 8 ng/mL)	2.396 ns	7.588 ns	5.545 ns	6.531 ns

Unstandardised β coefficients.

eGFR = estimated glomerular filtration rate, ns = nonsignificant.

* *P* < .05, Model fit (*R*^2^): 1 mo = 0.346; 3 mo = 0.487; 6 mo = 0.475; 12 mo = 0.395. Analytic sample sizes: 1 month, n = 149; 3 months, n = 149; 6 months, n = 149; 12 months, n = 149. (1 & 12 months: linear regression; 3 and 6 months: robust regression.)

** *P* < .01.

*** *P* < .001.

## 4. Discussion

The clinical implications of early tacrolimus fluctuations remain incompletely understood. Tacrolimus is an immunosuppressive drug with a narrow therapeutic window; overdose can cause toxicity, while underdose can trigger rejection.^[25]^ The literature reports that high IPV is generally associated with poor graft outcomes.^[4,13,26-28]^ However, some studies have failed to find a significant association between tacrolimus IPV and 1-year eGFR.^[4,23,29]^ Such discrepancies have been attributed to methodological heterogeneity and varying observation durations.^[13]^ In our study, a decrease in GFR was observed at 3 months in the high variability group, but no significant difference was noted between the 2 groups at 1 and 12 months. This pattern indicates that early pharmacokinetic variability exerts minimal influence on short-term renal performance, while additional mechanisms may come into play for long-term outcomes.^[4,14]^

Maintaining tacrolimus levels within the target range is critically important during this period. In our study, more frequent occurrences of sub- and supra-therapeutic levels were observed in the high variability group. In the literature, tacrolimus episodes outside the target range have been shown to negatively affect graft survival.^[15,25,30]^ On the other hand, it has been reported that both acute rejection and graft loss are significantly reduced in patients with a high target range retention time.^[25,31]^ These findings support the utility of time-in-range metrics over simple mean concentration values in dose optimization.^[32-34]^ The subtherapeutic/supra-therapeutic differences observed in our study are consistent with these relationships.

Kidney recipient profile and donor characteristics are also important factors influencing graft function. In the present cohort, recipient age, donor age, and biopsy-proven graft dysfunction showed consistent negative associations with eGFR across follow-up. An increased proportion of living donors was observed among recipients with higher tacrolimus variability, a finding warranting further investigation. Recent data indicate that transplants from living donors provide higher graft function and survival in the long term compared with deceased-donor transplantation.^[35]^ However, in the present multivariable models, living donor status was associated with eGFR only in the 1-month model and did not show a consistent association thereafter. The literature also reports that male donor kidneys may contain greater nephron mass than female donor kidneys and may therefore improve graft function.^[36]^ In our cohort, male donor sex showed a positive association with eGFR, but this association reached statistical significance only at 6 months and should therefore be interpreted cautiously. Donor age showed a consistent negative association with eGFR at 1, 3, 6, and 12 months, suggesting that donor-related renal reserve may remain clinically relevant during the first posttransplant year. A recent investigation reported no statistically significant associations between donor demographics and long-term graft performance.^[37]^ Such divergence may reflect differences in cohort structure, follow-up duration, donor characteristics, immunosuppressive intensity, and perioperative management.

From an immunological perspective, in our study, biopsy-proven graft dysfunction was associated with poor eGFR at all time points. This finding is consistent with the previously reported view that chronic damage and immune response markers impair graft function. Transient rejection and DGF also tended to be associated with lower function; meta-analyses in the literature have shown that DGF significantly increases graft loss and acute rejection in the first year.^[38]^ In our study, the effect of DGF was only at the trend level (p ≈ 0.06). This discrepancy raises questions about the role of nuances in follow-up duration and immunosuppression protocols. While the role of acute rejection in long-term function is generally accepted, early rejection and effective treatment may not have created a significant difference in graft function in this study.^[14,38,39]^ Our findings align with prior reports linking high IPV and off-target concentrations with adverse graft outcomes, while also aligning with approaches suggesting that more complex interactions may be at play.

## 5. Conclusion

Variability in tacrolimus levels during the early posttransplant period was found to be significantly associated with increased frequencies of subtherapeutic and supratherapeutic measurements. Despite these deviations, no consistent deterioration in renal function was observed over the 1-year follow-up. This pattern suggests that early pharmacokinetic instability, while reflective of off-target exposure, may not independently predict medium-term graft performance.

## 6. Study limitations

Despite the structured assessment of intrapatient tacrolimus variability and its associations with renal performance, several methodological aspects may have influenced interpretability. The retrospective nature of data collection introduces a reliance on available clinical documentation, which may omit unrecorded confounders. Additionally, the study population was drawn from a single transplant center, potentially limiting external comparability due to institutional practices in monitoring or immunosuppressive adjustments.

## Author contributions

**Conceptualization:** Ferah Taran.

**Data curation:** Ferah Taran.

**Formal analysis:** Ferah Taran.

**Funding acquisition:** Ercan Türkmen.

**Methodology:** Ercan Türkmen.

**Project administration:** Ercan Türkmen.

**Resources:** Ferah Taran.

**Writing – original draft:** Ferah Taran.

**Writing – review & editing:** Ercan Türkmen.
